# The Impact of Physiotherapy Rehabilitation on Functional Recovery in Postoperative Paraplegia Following L2-L3 Laminectomy With Discectomy: A Case Report

**DOI:** 10.7759/cureus.110390

**Published:** 2026-06-07

**Authors:** Sakshi P Kavitake, Suraj Kanase

**Affiliations:** 1 Department of Neurosciences, Krishna College of Physiotherapy, Krishna Vishwa Vidyapeeth (Deemed to be University), Karad, IND

**Keywords:** lower limb paralysis, lumbar spine injury, paraplegia, physiotherapy, rehabilitation

## Abstract

Postoperative paraplegia is a severe neurological complication that significantly affects functional mobility and quality of life. This case report describes the rehabilitation course of a 72-year-old male who was diagnosed with paraplegia following an L2-L3 laminectomy and discectomy. The patient presented with a lack of movement in both lower limbs, bilateral foot drop, altered sensation over the left sole, and a reduced range of motion following surgery. An individualized physiotherapy rehabilitation program was designed after a detailed assessment and goal-setting. Rehabilitation primarily focused on bed mobility training, sensory re-education, range of motion exercises, strengthening exercises, and functional training. Additional interventions were aimed at preventing secondary complications and improving overall functional independence. Progress was regularly monitored throughout the rehabilitation period.

Following four weeks of rehabilitation, gradual improvements were observed in lower limb muscle activation, bed mobility, sitting balance, and functional mobility. The patient also demonstrated improvements in overall physical condition and independence in daily activities. This case report highlights the importance of early and structured physiotherapy rehabilitation in promoting functional recovery and improving quality of life in patients with postoperative paraplegia. Further documentation of similar cases may help develop effective rehabilitation strategies for neurological recovery following spinal surgery.

## Introduction

Postoperative paraplegia is a severe neurological complication that may occur following spinal surgeries, traumatic spinal injuries, or spinal cord compression. It results in significant motor, sensory, and functional impairments, thereby significantly affecting an individual’s mobility, independence, and quality of life [1]. Degenerative spinal disorders that cause chronic nerve root or spinal cord compression are among the common indications for decompressive spinal procedures such as laminectomy and discectomy [2]. Although these procedures are frequently performed to relieve pain and neurological symptoms, post-surgical neurological deficits may occasionally occur due to nerve root injury, vascular compromise, edema, or spinal cord damage [3].

Postoperative paraplegia, although relatively uncommon, represents one of the most devastating complications of spinal surgery, with reported incidence rates ranging from 0.2% to 1% following lumbar decompressive procedures. Its occurrence necessitates prompt and structured rehabilitation to maximize neurological and functional recovery. Paraplegia following spinal surgery can lead to lower limb weakness, sensory disturbances, impaired balance, reduced functional mobility, and dependence in activities of daily living. Prolonged immobility may further contribute to secondary complications such as joint stiffness, muscle atrophy, pressure sores, respiratory complications, and reduced cardiovascular endurance. Early identification and management of these impairments are therefore essential for improving recovery outcomes [4]. Physiotherapy plays a critical role in addressing these deficits and maximizing recovery potential through early, structured, and individualized intervention.

Physiotherapy rehabilitation plays a vital role in restoring functional mobility, improving muscle strength, preventing secondary complications, and enhancing overall independence in patients with neurological deficits [5]. A structured rehabilitation program that includes bed mobility training, sensory re-education, range of motion exercises, strengthening exercises, balance training, and functional task-oriented activities can significantly improve physical function and quality of life in paraplegic patients [6]. This case report describes the impact of physiotherapy rehabilitation on functional recovery in a 72-year-old male who developed paraplegia following L2-L3 laminectomy and discectomy. The report highlights the importance of early and individualized physiotherapy intervention for enhancing mobility and functional independence following postoperative neurological complications.

Written informed consent was obtained from the patient for publication of this case report and associated clinical information.

## Case presentation

Patient information

A 72-year-old male retired high school teacher from Karad presented with complaints of an absence of movement in both lower limbs, bilateral foot drop, and altered sensation over the left sole following an L2-L3 laminectomy and discectomy performed on September 4, 2025. The patient was right-hand dominant. He had experienced radiating pain in the right lower limb for the past five years, secondary to degenerative lumbar spine changes with nerve root involvement. Following surgical intervention, he developed paraplegia with significant functional limitations and was referred for physiotherapy rehabilitation. There was no significant past medical history of hypertension, diabetes mellitus, stroke, Parkinson's disease, or traumatic brain injury.

Clinical findings

On initial physiotherapy evaluation, the patient was conscious, oriented, and had stable vital signs. The patient was assessed in a supine position. Bilateral lower limb movements were absent, and bilateral foot drop was noted. Sensory deficits were present over the left sole. Functional assessment revealed severe limitation in bed mobility and complete dependence for activities of daily living. Muscle tone was reduced in the bilateral lower limb musculature, including the hip flexors, extensors, abductors, adductors, knee flexors and extensors, and ankle plantarflexors and dorsiflexors. Range of motion was restricted at the hip (flexion limited to approximately 60°), knee (flexion limited to approximately 70°), and ankle joints bilaterally, likely due to prolonged postoperative immobilization. Further neurological assessment, including Manual Muscle Testing (MMT) and reflex examination, was conducted to document baseline status.

Table 1 shows the timeline of the patient’s condition, surgical intervention, and physiotherapy rehabilitation process. Physiotherapy rehabilitation was initiated after surgery and continued progressively for four weeks.

**Table 1:** Chronological timeline of symptom onset, surgical intervention, and physiotherapy rehabilitation

Incident	Date
Onset of radiating pain in the right lower limb	For the past 5 years
Hospital admission	September 1, 2025
L2-L3 laminectomy and discectomy	September 4, 2025
Physiotherapy rehabilitation started	September 10, 2025
Duration of rehabilitation	4 weeks

Table 2 presents the baseline MMT findings of the lower limb muscles. Reduced muscle strength was observed bilaterally, with greater weakness on the left side. Complete absence of ankle movements was noted bilaterally, indicating severe motor impairment following surgery.

**Table 2:** Baseline MMT findings MMT of the upper and lower limbs of the patient. 2: Full ROM with gravity counterbalance; 1: flicker contraction MMT: Manual Muscle Testing; ROM: range of motion

Joint	Muscle	Right side	Left side
Hip	Flexor	2	1
	Extensor	2	1
	Abductor	2	1
	Adductor	2	1
Knee	Flexor	2	1
	Extensor	2	1
Ankle	Plantarflexor	0	0
	Dorsiflexor	0	0

Table 3 demonstrates the reflex examination findings. Superficial and deep tendon reflexes in the upper limbs were preserved bilaterally, while reduced knee reflexes and absent ankle and plantar reflexes indicated neurological involvement affecting the lower limbs.

**Table 3:** Reflexes 0: no reflex; +: diminished; ++: normal. For the plantar reflex, 0 indicates an absent response (neither flexor nor extensor response elicited)

Reflexes	Right side	Left side
Bicep	++	++
Tricep	++	++
Supinator	++	++
Knee	_+_	+
Ankle	0	0
Plantar	0	0

Diagnostic assessment 

The MRI findings are illustrated in Figure 1. The MRI was performed before surgical intervention on September 1, 2025, as part of the preoperative diagnostic workup, and the findings helped guide the decision for L2-L3 laminectomy and discectomy. MRI of the lumbosacral spine with whole-spine screening revealed loss of lumbar lordosis with multilevel degenerative (spondylotic) changes. Diffuse disc-osteophyte complexes were noted at multiple levels (L1-S1) with varying degrees of canal and foraminal narrowing. At the L2-L3 level, a diffuse disc-osteophytic bulge with posteroventral and right paracentral protrusion was seen compressing the thecal sac and the right traversing nerve root, with indentation of the left traversing and bilateral exiting nerve roots. Associated facet joint hypertrophy resulted in severe spinal canal stenosis at this level. Overall, MRI findings were consistent with multilevel lumbar spondylosis with severe canal stenosis at L2-L3, correlating with the patient’s postoperative neurological deficits and functional impairment.

**Figure 1:**
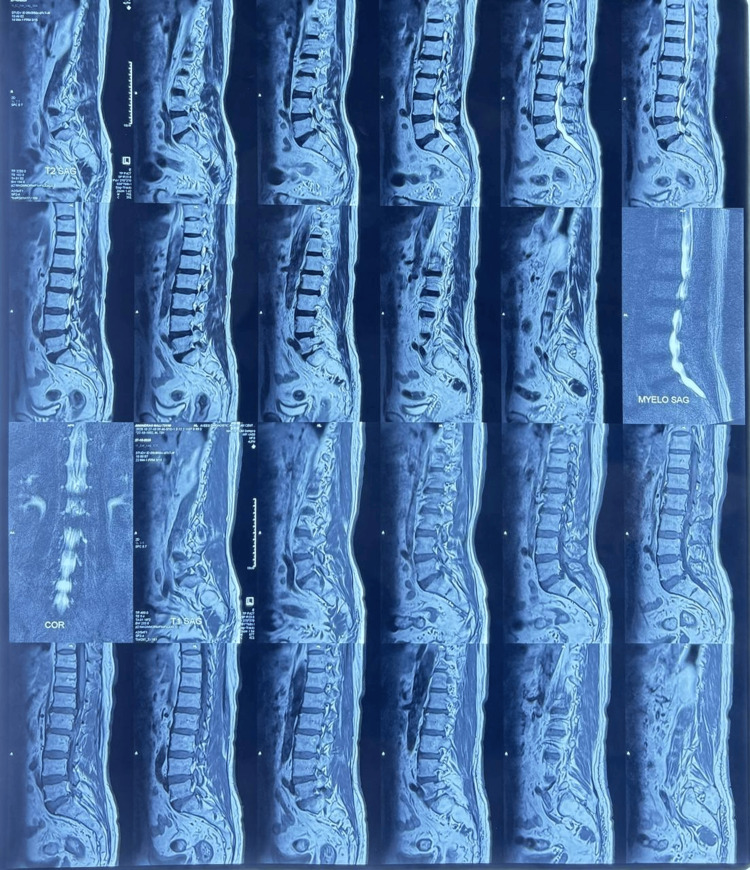
MRI lumbosacral spine with whole-spine screening The image shows multilevel degenerative lumbar spondylotic changes with severe spinal canal stenosis at L2-L3 due to diffuse disc-osteophytic bulge and facet joint hypertrophy MRI: magnetic resonance imaging

Postoperative pain management

Table 4 summarizes the medications prescribed to the patient during the postoperative period for pain management, neurological support, gastric protection, and overall recovery following spinal surgery.

**Table 4:** Postoperative medications prescribed Medications were for pain management, neurological support, gastric protection, and recovery following spinal surgery OD: once daily

Medication	Dosage	Duration
Tab. Gabapin NT	400 mg	OD
Tab. Deplatt A	150 mg	OD
Tab. Atore 20	-	OD
Tab. PAN	40 mg	OD
Tab. Ultracet	-	OD
Gel Anometrogyl	-	-
Tab. Nurewire	-	OD

Rehabilitation course

Table 5 outlines the week-wise goal-oriented physiotherapy rehabilitation protocol implemented for the patient. The rehabilitation program progressed systematically from prevention of secondary complications and respiratory care to mobility training, muscle strengthening, balance training, gait re-education, and functional independence according to the patient’s recovery status and tolerance.

**Table 5:** Week-wise goal-oriented physiotherapy rehabilitation protocol based on FITT principles implemented during the four-week rehabilitation period FIIT: Frequency, Intensity, Time, Type; DVT: deep vein thrombosis; ROM: range of motion

Week	Goal	Therapeutic intervention/treatment protocol
1	Patient education, prevention of secondary complications, and respiratory care	• Educated the patient and caregivers regarding the condition, prognosis, positioning, pressure relief, and importance of physiotherapy rehabilitation. • Bed mobility exercises and repositioning every two hours to prevent pressure sores. • Ankle-toe movements and passive lower limb exercises to prevent DVT. • Use of a pressure-relieving mattress (alternating air cell mattress) to minimize pressure-related complications. • Breathing exercises, including diaphragmatic breathing, pursed lip breathing, thoracic expansion exercises, and suctioning. • Bilateral lower limb passive ROM exercises. • Static stretching of the hamstring (supine straight leg raise stretch position) and the tendo Achilles (supine ankle dorsiflexion stretch), with 15-second hold for three repetitions each, performed once daily
2	Improve mobility and initiate muscle activation	• Supine to side-lying transitions, bed rolling, and side sitting activities. • Active-assisted ROM exercises for upper and lower limbs (10 repetitions × three sets). • Isometric (static) muscles strengthening exercises for bilateral upper and lower limbs, targeting hip flexors, extensors, abductors, adductors, knee flexors, and extensors (10-second hold × 10 repetitions × three sets). • Pelvic floor strengthening using Kegel’s exercises and transverse abdominal contractions. • Hip abductor and adductor exercises. • Sensory integration therapy applied to the bilateral lower limbs (plantar surface of foot, dorsum of foot, lower leg, and thigh) using textures including cotton cloth, silk cloth, Turkish cloth, feathers, and sand, progressing from distal (plantar surface) to proximal (thigh) to facilitate sensory re-education. • Sitting balance exercises with support
3	Improve trunk control, balance, and strength	• Posterior pelvic tilting, pelvic rotations, and pelvic bridging exercises. • Shoulder protraction and retraction exercises. • Static and isometric strengthening exercises, including low squats, split squats, static lunges, leg extensions, and lateral shoulder raises (10 repetitions × three sets). • Static sitting balance exercises, including supported sitting with progressive reduction of support, lateral and anterior reach-out, perturbation training, and vestibular ball sitting-eyes open and closed (20-second hold × three repetitions × two sets). • Weight shifting exercises in sitting and standing positions. • Continued stretching and sensory integration therapy. Continued sensory integration therapy with progression to moderate and rough textures (Turkish cloth, sand), eyes open and closed alternated (30 seconds per area, 10-15 minutes per session), combined with proprioceptive input through weight-bearing activities
4	Improve gait, coordination, endurance, and functional independence	• Gait training 10-15 minutes progressing toward independent ambulation. • Frenkel's exercises in standing positions (10 repetitions × two sets). • Tandem walking (3 × 10 steps) and coordination exercises. • Standing balance training and dynamic postural activities. • Functional mobility training, including transfers and ADL. • Continued strengthening, respiratory exercises, and pelvic floor exercises. • Sensory integration therapy continued with combined tactile and proprioceptive stimulation during standing and functional weight-bearing tasks to consolidate sensory-motor integration (10-15 minutes per session).• Home exercise program prescribed and demonstrated to patient and caregiver, including: passive and active-assisted ROM exercises for bilateral lower limbs (10 repetitions × 2 sets), ankle pump exercises (15 repetitions × two sets), isometric strengthening of hip and knee musculature (10-second hold × 10 repetitions), breathing exercises (diaphragmatic breathing, 10 repetitions), and pressure relief repositioning every two hours. The caregiver was educated on correct technique, safety precautions, skin inspection, and signs requiring medical attention. Exercises to be performed once daily

Rehabilitation Protocol Overview (FITT Principles)

Frequency: Once daily, six days per week.

Intensity: Progressed weekly based on patient tolerance and MMT grade improvement.

Time: 45-60 minutes per session.

Type: Progressive, goal-oriented physiotherapy as outlined below

Physiotherapy was initiated on Day six post-surgery (September 10, 2025), once the patient was clinically stable and cleared by the surgical team. All exercises in Week one commenced from Day one of physiotherapy. Progression to subsequent weeks was determined by clinical response, muscle activation, and patient tolerance.

Table 6 demonstrates the progressive improvement in the patient’s muscle strength and functional independence over the four-week rehabilitation period. Outcome assessment using MMT and Functional Independence Measure (FIM) indicated gradual recovery in lower limb muscle activation and performance of daily functional activities following structured physiotherapy rehabilitation. 

**Table 6:** Progressive changes in muscle strength and functional independence measured using MMT and FIM during rehabilitation MMT: Manual Muscle Testing; FIM: Functional Independence Measure

Sl. No.	Outcome measure	Week 1	Week 2	Week 3	Week 4
1	MMT	2/5	2+/5	3/5	3+/5
2	FIM	1/7	3/7	5/7	6/7

## Discussion

Postoperative paraplegia following lumbar spine surgery is a serious neurological complication that significantly affects mobility, functional independence, and quality of life [7]. Although procedures such as laminectomy and discectomy are commonly performed to relieve neural compression, postoperative neurological deficits may occur due to nerve injury, vascular compromise, edema, or inflammation [8]. These complications can result in severe motor weakness, sensory impairment, impaired balance, and dependence in activities of daily living [9].

Early physiotherapy rehabilitation plays a vital role in improving functional outcomes in patients with postoperative paraplegia [10]. In the present case, a structured and progressive rehabilitation program resulted in gradual improvements in lower limb muscle activation, trunk control, sitting balance, standing tolerance, and assisted ambulation over four weeks. The rehabilitation protocol was designed based on the patient’s clinical condition and focused on addressing motor weakness, sensory deficits, impaired mobility, and reduced functional independence.

Physiotherapy management primarily focused on the prevention of secondary complications associated with prolonged immobilization, including muscle atrophy, joint stiffness, pressure sores, and decreased cardiovascular endurance [11-13]. Interventions such as positioning, respiratory exercises, range of motion exercises, sensory re-education, strengthening exercises, trunk stabilization, balance training, and functional mobility exercises were incorporated to facilitate neurological recovery and improve independence [5,14,15]. It should also be acknowledged that spontaneous neurological recovery following surgical decompression of the spinal cord may have contributed to the functional improvements observed. The decompression procedure itself relieves mechanical compression and reduces ischemic injury, which may facilitate some degree of intrinsic neural recovery independent of physiotherapy. The relative contributions of surgical decompression and physiotherapy rehabilitation to the overall outcome cannot be fully distinguished in a single case report design.

The improvements observed in this case may be attributed to early mobilization and repetitive task-specific training, which are known to promote neuroplasticity and motor relearning in neurological rehabilitation [16,17]. Sensory integration therapy and progressive functional training may also have contributed to improved proprioception, muscle activation, and postural control. Continuous monitoring and progression of exercises according to patient tolerance further supported functional recovery [18,19].

Outcome assessment using MMT and FIM demonstrated progressive improvement in muscle strength and functional independence throughout the rehabilitation period. These findings highlight the importance of individualized and goal-oriented physiotherapy rehabilitation in enhancing recovery following postoperative paraplegia. Despite the improvements achieved, recovery following postoperative paraplegia remains variable depending on the severity of neural involvement, timing of intervention, and patient-specific factors. Further studies and documentation of similar cases are necessary to develop evidence-based rehabilitation protocols for neurological recovery following spinal surgery.

Limitations and future directions

This case report is limited by its single-patient design, which restricts the generalizability of the findings. The absence of a control group makes it difficult to determine the independent contribution of physiotherapy rehabilitation to the observed improvements. In addition, long-term follow-up was not available to assess the sustainability of functional recovery. Future research involving larger sample sizes, longer follow-up periods, and controlled study designs is needed to establish evidence-based physiotherapy rehabilitation protocols for patients with postoperative paraplegia following spinal surgery.

## Conclusions

This case report underscores the crucial role of physiotherapy rehabilitation in improving functional recovery in a patient with postoperative paraplegia following L2-L3 laminectomy and discectomy. By addressing the multiple impairments associated with neurological deficits through a structured and progressive rehabilitation program, significant improvements were observed in muscle activation, balance, mobility, and functional independence. The report highlights the importance of early intervention, task-specific training, and a multidisciplinary, patient-centred approach in enhancing recovery and minimizing secondary complications following spinal surgery. Furthermore, this report emphasizes the need for continued research, clinical collaboration, and development of evidence-based rehabilitation protocols for patients with postoperative neurological complications.
